# Correspondence

**Published:** 1958-01

**Authors:** Joseph Sluglett


### *Correspondence*

DEAR SIR,

I would like to express my appreciation of Professor Bruce Perry's lecture on the medical curriculum; it was so full of good sense. Many of us in General Practice are interested in medical education, and about a year or so ago two senior medical students were invited to address the Bristol South Medical Group on that subject. I was interested to learn that matters were very little different from my student days, indeed to all the "ologies" we had to cope with 25 years ago many more have been added, as Professor Bruce Perry put it there has been no change only more overloading.

What surprises me most and, as he admitted, puzzled him too is that, despite the wealth of writing on the subject over the past years, very little has been achieved in the way of reforming the curriculum and easing the burden of the student. Of course it may well be that in the teaching hospitals and universities there are difficulties to contend with just as we have, people who are impervious to new ideas and extremely resistant to the suggestion that there might after all be better ways of teaching students.

I liked the reference to the American tutorial system whereby discussion rather than lectures is the method of imparting clinical experience but this he thought impossible here because of staff difficulties. In my clinical days in Glasgow each teaching unit at the Western Infirmary had on its staff one or two G.P.'s in practice in the City who attended several mornings a week to act as tutors, and this worked very well in practice.

The G.P. student training scheme has been in operation in Bristol now for some years and is much appreciated by the students, but I feel it is inadequate and applied far too late in the curriculum; we could do far more to our mutual benefit. I think it is true to say that after some years experience in the practice of the art and science of medicine our attitude to our patients is conditioned very largely by the atmosphere in which we work. Up until quite recently medical students have been instructed almost exclusively by teachers whose clinical experience has for the most part been gained in hospital dealing with patients referred there by General Practitioners. Surely the General Practitioner with his experience derived from being the first to see the patient and deal with him in the background of his home and working condition has a part to play in a reformed medical curriculum.

Yours sincerely,

JOSEPH SLUGLETT.

